# Optimizing the Thermal Read-Out Technique for MIP-Based Biomimetic Sensors: Towards Nanomolar Detection Limits

**DOI:** 10.3390/s130709148

**Published:** 2013-07-16

**Authors:** Bram Geerets, Marloes Peeters, Bart van Grinsven, Karolien Bers, Ward de Ceuninck, Patrick Wagner

**Affiliations:** 1 Institute for Materials Research, Hasselt University, Wetenschapspark 1, B-3590 Diepenbeek, Belgium; E-Mails: bram.geerets@student.uhasselt.be (B.G.); bart.vangrinsven@uhasselt.be (B.G.); karolien.bers@uhasselt.be (K.B.); ward.deceuninck@uhasselt.be (W.D.C.); patrick.wagner@uhasselt.be (P.W.); 2 IMEC vzw, Division IMOMEC, Wetenschapspark 1, B-3590 Diepenbeek, Belgium

**Keywords:** heat-transfer method (HTM), molecularly imprinted polymers (MIPs), L-nicotine, PID parameters

## Abstract

In previous work, the novel heat-transfer method (HTM) for the detection of small molecules with Molecularly Imprinted Polymers (MIP)-type receptors was presented. In this study we focus on optimization of this sensor performance, with as final aim to lower the detection limit by reducing the noise level. It was determined that the noise originates foremost from the power supply, which can be controlled by varying the PID parameters. Therefore, the effect of the individual parameters was evaluated by tuning P, I and D separately at a temperature of 37 °C, giving a first indication of the optimal configuration. Next, a temperature profile was programmed and the standard deviation of the heat-transfer resistance over the entire regime was studied for a set of parameters. The optimal configuration, P1-I6-D0, reduced the noise level with nearly a factor of three compared to the original parameters of P10-I5-D0. With the optimized settings, the detection of L-nicotine in buffer solutions was studied and the detection limit improved significantly from 100 nM to 35 nM. Summarizing, optimization of the PID parameters and thereby improving the detection limit is a key parameter for first applications of the HTM-method for MIP receptors in analytical research.

## 1. Introduction

**Figure 1.**
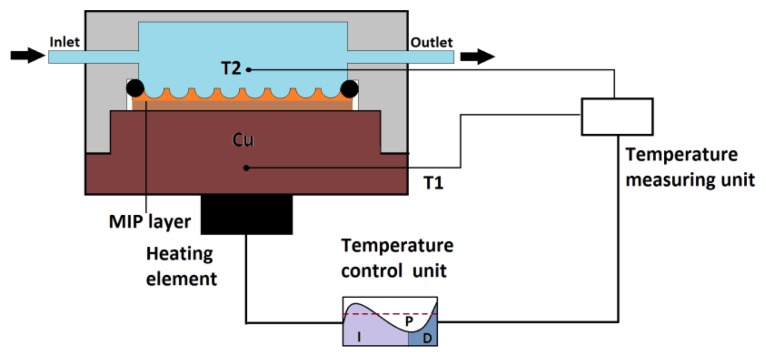
Shows schematically the experimental setup. The temperature of the copper block (*T*_1_) is actively steered *via* the temperature control unit through the heating element while *T*_2_, the temperature of the fluid, is solely monitored. For the measurements the power is also a significant factor, which is controlled by the PID-element [20].

Molecularly Imprinted Polymers (MIPs) are synthetic materials which mimic the recognition and binding behavior of natural antibodies [1–3]. These tailor-made receptors are highly selective, stable, and resistant to a wide range of pH, solvents and temperature [4,5]. In comparison to natural receptors, MIPs are cheap due to their straightforward synthesis [6]. These properties make them an interesting tool for different application areas, including separation science and purification [7], biosensors [8], catalysis and drug delivery [9,10]. For chromatographic purposes, MIPs can be readily used by packing them directly into separation columns [7]. However, the integration of MIPs into sensing devices remains challenging. The majority of the sensor platforms is based on gravimetric detection [11–14] and electrochemical techniques [15–19]. Gravimetric detection is laborious, while the analysis with electrochemical techniques is often complicated. Van Grinsven *et al.* proposed a technique based on heat-transfer resistance to detect single nucleotide polymorphisms in DNA [20]. Recently, a similar approach was employed for signaling molecules with MIP-type receptors [21]. This heat-transfer method (HTM) requires only two thermocouples, a proportional-integral-derivative (PID) controller and an adjustable heat source, eliminating the need of sophisticated equipment. For proof-of-principle purposes, the response of a MIP to increasing L-nicotine concentrations in buffer solutions was studied and a detection limit of 100 nM in buffer solutions was achieved. This is comparable to electrochemical impedance spectroscopy which was used as reference technique. A schematic design of this setup is shown in Figure 1.

In this manuscript we will focus on optimization of the sensor performance, with the aim to lower the detection limit. To this end, the noise level should be reduced since the detection limit corresponds to the concentration where the signal is three times the standard deviation. In order to control the temperature of the copper block, an algorithm is combined with a feedback loop. This algorithm functions as a set point controller. The controller implements the proportional value as the present error, where the integral and derivative correspond to the average of the past errors and the prediction of future errors respectively [22,23]. The effect of the temperature and the PID parameters of the control unit on the noise level are analyzed. As a starting point, the parameters of previous research (P = 10, I = 5, D = 0) are used [20,21]. These parameters are selected since they ensure high temperature control, with a minimal uncertainty of 0.02 °C from the set temperature. However, for the measurements not solely the temperature is studied, but the heat-transfer resistance which also involves the power. The observed noise in the experiments is mainly due to changes in the voltage, necessary to keep the temperature of the copper constant. In this manuscript other PID parameters are tested systematically and evaluated if they allow a better tradeoff in temperature and power control, resulting in a lower noise ratio of the overall heat-transfer resistance.

Using the optimized PID parameters, the noise level is shown to reduce nearly threefold, lowering the detection limit to 35 nM. This is well within the physiologically relevant range of L-nicotine, since salivary and urinary concentrations nicotine are in the micromolar regime, 0.2-1,000 μM in saliva and 0.3-10 μM in urine [24,25]. Therefore, there is no direct need to further optimize the sensor performance for the detection of this template. However, it should be considered that this read-out technique can also be employed for other targets that are of higher interest for biomedical research, for instance histamine and serotonin. We will demonstrate that optimization of the PID parameters is a key element for future measurements in biological samples [26,27]. Summarizing, the HTM method enables fast and low-cost measurements and optimizing the sensor performance is an important step for real analytical applications.

## 2. Experimental Section

### 2.1. Design of the Sensor Setup

In Figure 1 the sensor platform is presented. The MIP substrates are mounted horizontally into a home-made Perspex flow cell with an internal volume of 110 μL. The sensor surface is sealed off from the external environment using an O-ring with an area of 28 mm^2^. The substrate was attached to the copper block with silver paste, ensuring good thermal contact. On top of the copper block, the power resistor (MHP TO-220, 20 W, Farnell, Grace-Hollogne, Belgium) with a prefixed resistance of 22 Ω, is attached which is connected to the temperature control unit, enabling control of the copper temperature. In order to deliver a specific output voltage to our power resistor, a proportional integral derivative (PID) controller is utilized. This control unit will calculate the necessary output voltage to approach the determined temperature set point. Therefore, two miniaturized thermocouples (Type K, 500 μm, TC Direct, Nederweert, The Netherlands) are used to obtain, respectively, the copper (*T*_1_) and temperature in the fluid (*T*_2_). The first thermocouple (*T*_1_) is placed 4 mm inside the copper block, while the second thermocouple (*T*_2_) is positioned in the flow cell 1.7 mm above the sensor surface in the liquid. The measured temperatures are dispatched to a data acquisition unit (Picolog, TC08, Picotech, Cambridgeshire, UK) and forwarded to the PID controller. The PID controller compares the prefixed temperature set point and the actual measured temperature and subsequently determines the output voltage towards the power resistor. The output voltage is directed through a second controller (NI USB 9263, National Instruments, Austin, TX, USA) to a power operational amplifier (LM675, Farnell), before arriving at the power resistor. The setup is equipped with a tubing system which makes it possible to connect it to a programmable syringe pumping system (ProSense, Ne-500, Ede, The Netherlands). Additionally, the flow cell contains a gold wire which can be employed as an electrode for impedance measurements [20,28].

### 2.2. Optimizing the PID Settings

The measurements to study the PID settings are performed on a blank aluminum substrate with an area of 1 cm^2^ and a flow cell filled with 1× phosphate buffered saline (PBS) of pH 7.4. The temperature dependence of the PID parameters is evaluated and the optimal settings are determined by analyzing the standard deviation on the signal. The parameters settings with the lowest standard deviation are marked as the optimal configuration. A temperature profile is programmed which consists of, respectively, plateaus and ramps and is in the temperature range of 30–85 ° C at an ambient temperature of 19.00 °C. The temperature plateaus last 20 min, after which the temperature is raised in a controlled fashion with 1 °C/min for 5 min to reach the next plateau. The time in which the temperature is increased will be referred to as the ramping phase. The *R_th_* (heat-transfer resistance) is calculated by dividing the difference in temperature between the copper (*T*_1_) and the fluid (*T*_2_) by the power needed to keep the copper at a constant temperature (Equation (1)). The power is obtained by dividing the square of the output voltage by the resistance of the power resistor [20]:

$$R_{th}=\frac{T_{1}-T_{2}}{P}$$

The standard deviation on the *R_th_* signal was calculated for the plateaus and the ramping phase. For the ramping phase, the *R_th_* signal is fitted with a linear fit. Subsequently, this linear fit is subtracted from the *R_th_* signal, thereby creating an artificially plateau out of a ramping phase. The standard deviation of this section was calculated over 300 points.

### 2.3. Preparation of the MIP Electrode

The synthesis procedure for the MIP and its reference, the non-imprinted polymer (NIP) for L-nicotine are described in detail in [8]. To prepare the functionalized electrodes, 1 × 1 cm^2^ aluminum electrodes are spincoated with conductive OC_1_C_10_-polyphenylenevinylene (PPV). This PPV derivative, serving as an adhesive layer, is synthesized via the sulfinyl precursor route [29]. MIP and NIP particles are applied to the surface with a polydimethylsiloxane (PDMS) stamp. Subsequently, the layer is heated to 120 °C, allowing the powder to partially sink into the MDMO-PPV layer. After cooling the substrates are washed with isopropanol, ensuring a strong fixation into the layer. The substrates are attached to the copper block, which is kept constant at 37.00 ± 0.02 °C. After stabilizing in 1× PBS, the electrodes are exposed to increasing concentrations of L-nicotine (10–1,000 nM) in order to determine the detection limit of the sensor platform.

## 3. Results and Discussion

### 3.1. Optimization of the Sensor Setup

**Figure 2.**
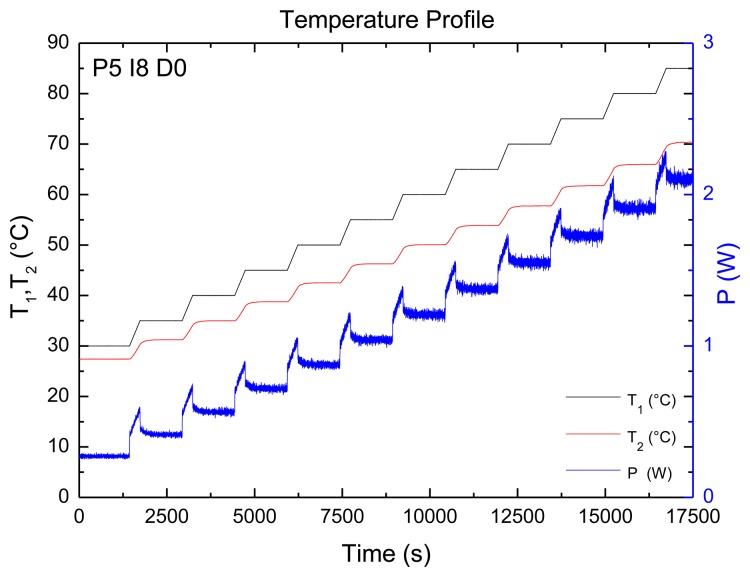
The temperature profile, used to study the noise level, consists of the copper temperature, *T*_1_ (black), the fluid temperature, *T*_2_ (red) and the power applied to the heating element (blue). The profile comprises plateau phases and ramping phases and is presented with a PID configuration of P5-I8-D0.

The blank aluminum substrates are mounted into the Perspex flow cell filled with 1× PBS. In order to study the temperature dependence of the PID settings, a temperature profile was designed in a regime of 30–85 °C containing intermediate stages every 5 ° C. This is shown in Figure 2. The black curve represents the temperature of *T*_1_, the red line demonstrates *T*_2_, and the blue curve stands for the applied power necessary for maintaining the copper temperature at each plateau. The PID settings to compute the representing figure were P5-I8-D0.

In order to determine the origin of the noise, the temperature signal was evaluated foremost. The noise of the temperature signal was determined to be 0.02 °C for the entire temperature range (30–85 ° C). Simultaneously, the power signal was examined where something striking was observed. There was a considerable amount of noise on the power signal, though this was absent in the temperature signal. Hereby, a first indication for optimization of the measuring signal was reflected and it is feasible to optimize the system by reducing the noise of the power supply.

**Figure 3.**
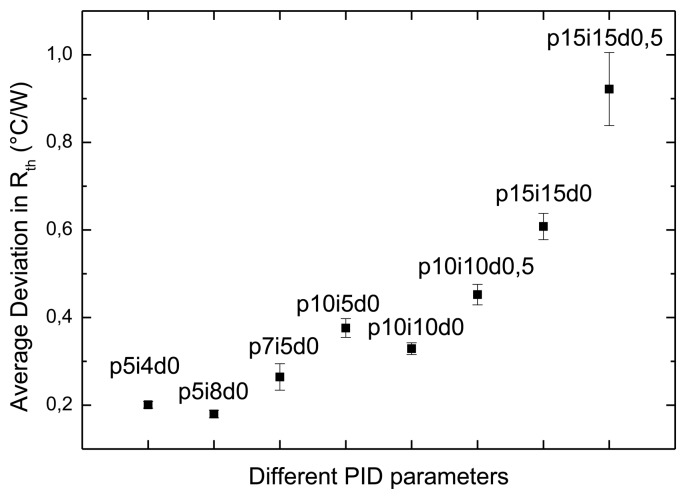
Ten different PID-parameters were examined in a wide regime of configurations at a stable temperature of 37.00 ± 0.02 ° C. The average uncertainty in *R_th_* (heat-transfer resistance) is plotted versus the different PID parameters.

Therefore, ten distinct different PID configurations were examined at a stable temperature of 37.00 ± 0.02 °C, which are shown in Figure 3. This enabled us to determine the effect of different values for the PID parameters. For instance, a PID configuration of P15-I15-D0.5 resulted in an average uncertainty in *R_th_*) of 0.921 ± 0.083 °C/W, where PID parameters of P5-I4-D0 result in an average uncertainty of only 0.201 ± 0.007 °C/W. The results of this first PID related effect implied that by omitting the derivative parameter, the noise level is reduced. These first implications enabled us to demarcate a specific range of PID configurations in order to examine further noise reduction. These configurations are shown in Table 1. To examine the temperature dependency of the PID settings, the above presented temperature profile was applied for every individual PID configuration.

The PID parameters are displayed in Table 1, giving a clear representation of the effect of each individual parameter. Moreover, we still differentiate between plateau phases and ramping phases. However, the representing numbers are averaged out for the entire temperature range and the error number for one PID configuration is rendered in one number. Hereby, it is possible to consider the effect of making small changes in the ratio of the PID parameters. We noticed that PID settings with low proportionality values only improve the signal-to-noise ratio of the heat transfer resistance signal when integral action has a relatively high value. A higher integral action will also guarantee a more stable signal. The derivative term was left out of consideration, due the results of the earlier performed experiment.

**Figure 4.**
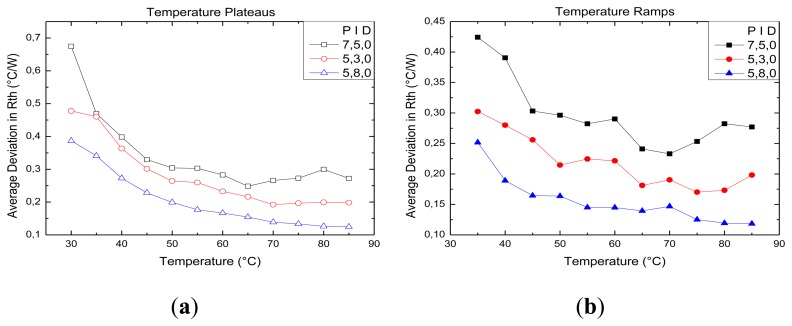
The average uncertainty of the thermal resistance was measured for the entire temperature range. This figure illustrates the average uncertainty in *R_th_* for every temperature plateau (**a**), and temperature ramp (**b**) for three selected PID configurations. The solid lines are a guide to the eye.

Figure 4a,b illustrate the averaged uncertainties of the heat transfer signal at specific temperatures levels for the plateau phases and the ramping phases. Three PID configurations were selected to illustrate the effect of changing the ratio between proportional and integral action. The following PID parameters were used P7-I5-D0, P5-I3-D0, and P5-I8-D0 to give a representative view of the deviation on the thermal resistance signal. As we can see, minor changes in configuration already result in a strong decrease in the average uncertainty of the HTM signal. Table 1 shows that P1-I6-D0 corresponds to the optimal configuration with an average uncertainty of only 0.095 ± 0.058 °C/W for plateau phases and 0.076 ± 0.026 °C/W for ramping phases. In comparison with the prior PID configuration (P10-I5-D0), this results in a 3-fold noise reduction. Furthermore, there is a difference in noise level between plateau and ramping phases. In order to maintain the plateau temperature, the heating element is switched on and off repetitively. This phenomenon results in a more alternating signal. However, due to the optimizing the PID configurations, this effect is minimized. In comparison with the ramping section, here the temperature of the copper is increased at a rate of 1 °C/min. Therefore, the power applied to the heating element is also increasing resulting in less error on the thermal resistance signal. Next to the effect of altering the PID parameters, we also noticed a temperature related effect. At elevated temperatures, the uncertainty of the thermal resistance becomes significantly reduced. The diminishing effect on the signal by increasing the temperature can be elucidated as an effect of heat transfer from the copper not only to the flow cell, but also towards the environment. At elevated temperatures this effect increases tremendously, resulting in an even more stable signal.

**Figure 5.**
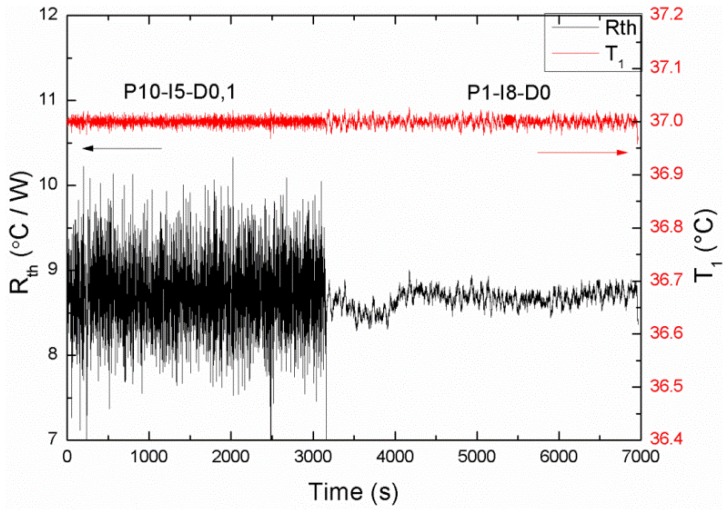
The *R_th_* measured in time for PID configurations P10-I5-D0,1 and P1-I8-D0, thereby directly showing the lowering of the noise by optimizing the PID parameters.

To summarize these results, in Figure 5 the *R_th_* in time is shown for the previous parameters (P10-I5-D0,1) and the optimized ones (P1-I8-D0).

### 3.2. Detection Limit

**Figure 6.**
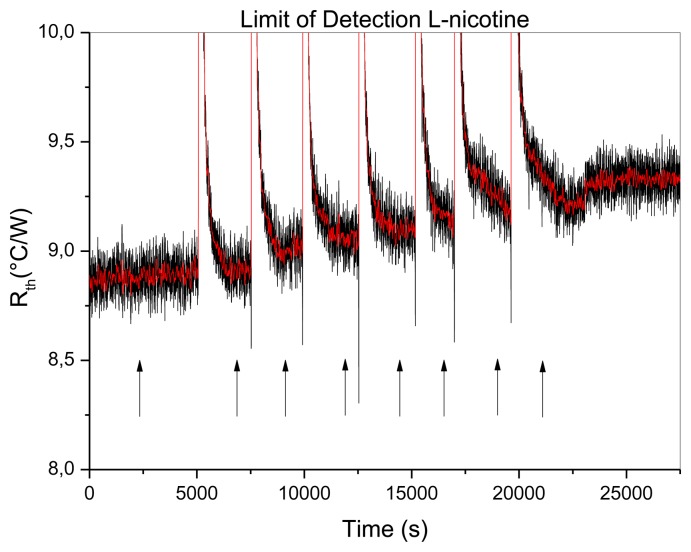
The measurement was performed with the optimized PID parameters of P = 1, I = 8 and D = 0. The Figure shows the response in thermal resistance upon increasing concentrations of L-nicotine (10, 25, 50, 100, 250, 1,000 nM). The additions are indicated by the arrows. The raw data was filtered with a percentile filter (50%) over 50 points, resulting in the smoothed red line.

In order to prove the lowering of the detection limit by reducing the noise, the detection of L-nicotine with MIPs was studied. In previous work by Peeters *et al.* [21], this detection limit was calculated to be 100 nM. To provide evidence of the influence of the PID configuration on HTM, concentrations are added in the range of 10-1,000 nM L-nicotine in PBS. The data of these additions is represented in Figure 6.

**Figure 7.**
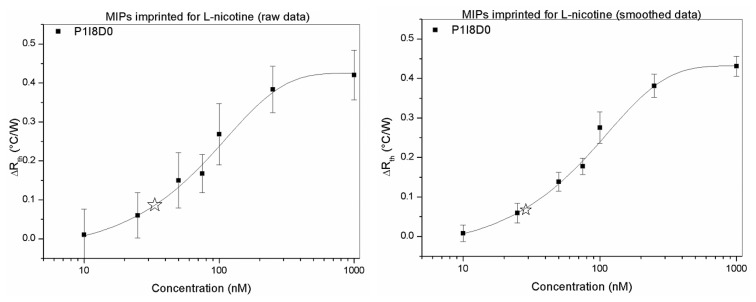
Dose-response curve of the MIP imprinted for L-nicotine for the raw and smoothed data. The difference in *R_th_* is plotted against logarithmic presented concentration. The concentration of L-nicotine varies in a range from 10 nM to 1 μM. The mathematical detection limit is illustrated by the asterisk, which is 35 nM for the raw data en 29 nM for the smoothed data.

The baseline of the *R_th_* was stipulated by initiating the measurement in PBS buffer solution, and stabilized at a *R_th_* value of 8.92 ± 0.04 °C/W. Subsequently, the addition of different concentrations L-nicotine was performed with increasing quantities, comprehending a stabilization period of at least 15 min. After each addition, an increase is noticed in *R_th_*. The addition of 1 μM L-nicotine resulted in a *R_th_* value of 9.34 ± 0.06 °C/W, resulting in an effect size of 4.4%. This is significantly higher compared to the size of the noise (0.04%). The difference in *R_th_* is calculated by deducting the baseline from the heat-transfer signal for each applied concentration and plotted in a dose-response curve. These results are summarized in Figure 7.

The dose response curve was constructed for a concentration range of 10 nM-1 μM. The standard deviation of the baseline was calculated to be 0.04 °C/W and was used to estimate the detection limit of the electrode, which was set to be three times the standard deviation of the baseline. This resulted in experimental detectable concentrations between 50 nM (Δ*R_th_* = 0.15 ± 0.07 °C/W) and 1 μM (Δ*R_th_* = 0.42 ± 0.06 °C/W). Additionally, the theoretical detection limit was estimated using a linear fit (*R*^2^ = 0.95) through the dose-response curve. The calculated detection limit was determined to be 35 nM, which lowers it a threefold compared to the results in [21]. The limit of quantification, equal to five times the standard deviation, corresponds to 70 nM. The same procedure was applied for the smoothed data, resulting in a detection limit of 29 nM and a limit of quantification of 58 nM.

## 4. Conclusions/Outlook

In this study, we presented the influence of PID parameters on the detection limit for small molecules with MIPs, using HTM as analytical technique. In previous work, Peeters *et al.* [21] showed a target concentration detection limit of 100 nM. These results were obtained with PID parameters of P10-I5-D0, which have a unfavorable high noise level (average uncertainty in *R_th_* = 0.698 ± 0.150 °C/W for plateaus and, respectively, 0.627 ± 0.097 °C/W for ramps. The origin of the noise was located to be in the power signal by separately analyzing the temperature and power data. The applied power is controlled via the PID parameters of the PID control unit. The optimal PID configuration, which results in the lowest uncertainty in *R_th_*, was calculated in a range from 30–85 °C differentiating between temperature plateaus and ramps. This is determined to be P1-I6-D0, with an average uncertainty in *R_th_* = 0.095 ± 0.058 °C/W for plateaus and 0.076 ± 0.026 °C/W for ramps, reducing the noise level by nearly a factor of three. Subsequently, the detection of small molecules with MIPs was re-examined using the optimized PID parameters. These resulted in a detection limit (35 nM) which is a threefold lower compared to the limit of detection obtained in previous experiments (100 nM). Other biogenic amines, such as histamine and serotonin, have significantly lower physiologically relevant concentrations. With the same setup and impedimetric detection, Horemans *et al.* [27] documented the presence of histamine in blood to be in a range of 10–1,000 nM and in a regime of 200–750 nM in urine. Peeters *et al.* [21] discovered a presence of 10–1,500 nM of serotonin in blood. The lowered noise level is therefore required to use HTM for performing measurements in biological samples. Summarizing, the improved signal-to-noise ratio of HTM enables detecting small molecules in the nanomolar range using MIP receptors, which makes it a promising technique for bioanalytical purposes.

## Figures and Tables

**Table 1.** The PID parameters shown in this table were selected after demarcating a specific regime. Hereby, it was possible to sharpen the PID parameters to be used. The first column gives a representation of the used PID configurations. The numbers illustrate respectively the proportional, integral, and derivative factors. The second and third column act as the calculated average uncertainty in *R_th_* for plateau phases and ramping phases for the entire temperature range.

**PID Configurations**	**Average Uncertainties in*R****_th_***(°C/W)**	

	**Plateau Phases**	**Ramping Phases**
1-4-0	0.137 ± 0.073	0.105 ± 0.046
1-6-0	0.095 ± 0.058	0.076 ± 0.026
1-8-0	0.101 ± 0.070	0.085 ± 0.022
1-10-0	0.107 ± 0.056	0.081 ± 0.036
3-4-0	0.130 ± 0.077	0.103 ± 0.038
3-6-0	0.153 ± 0.087	0.120 ± 0.047
3-8-0	0.123 ± 0.063	0.093 ± 0.042
3-10-0	0.111 ± 0.069	0.090 ± 0.030
5-4-0	0.168 ± 0.073	0.121 ± 0.067
5-6-0	0.156 ± 0.070	0.113 ± 0.061
5-8-0	0.165 ± 0.086	0.125 ± 0.056
5-10-0	0.144 ± 0.079	0.112 ± 0.046
5-15-0	0.135 ± 0.075	0.105 ± 0.042
10-5-0	0.698 ± 0.150	0.627 ± 0.097
